# Low cost satellite constellations for nearly continuous global coverage

**DOI:** 10.1038/s41467-019-13865-0

**Published:** 2020-01-10

**Authors:** Lake A. Singh, William R. Whittecar, Marc D. DiPrinzio, Jonathan D. Herman, Matthew P. Ferringer, Patrick M. Reed

**Affiliations:** 1 GEOINT Innovations Office, The Aerospace Corporation, Chantilly, VA 20151 USA; 2 Performance Modeling and Analysis Department, The Aerospace Corporation, Chantilly, VA 20151 USA; 3 Mission Analysis and Operations Department, The Aerospace Corporation, Chantilly, VA 20151 USA; 4 Civil and Environmental Engineering, University of California, Davis, CA 95616 USA; 5 National Geospatial Programs, The Aerospace Corporation, Chantilly, VA 20151 USA; 6 Civil and Environmental Engineering, Cornell University, Ithaca, NY 14853 USA

**Keywords:** Inner planets, Devices for energy harvesting, Aerospace engineering

## Abstract

Satellite services are fundamental to the global economy, and their design reflects a tradeoff between coverage and cost. Here, we report the discovery of two alternative 4-satellite constellations with 24- and 48-hour periods, both of which attain nearly continuous global coverage. The 4-satellite constellations harness energy from nonlinear orbital perturbation forces (e.g., Earth’s geopotential, gravitational effects of the sun and moon, and solar radiation pressure) to reduce their propellant and maintenance costs. Our findings demonstrate that small sacrifices in global coverage at user-specified longitudes allow operationally viable constellations with significantly reduced mass-to-orbit costs and increased design life. The 24-hour period constellation reduces the overall required vehicle mass budget for propellant by approximately 60% compared to a geostationary Earth orbit constellation with similar coverage over typical satellite lifetimes. Mass savings of this magnitude permit the use of less expensive launch vehicles, installation of additional instruments, and substantially improved mission life.

## Abstract (web summary)

Telecommunication, navigation and remote sensing services are highly dependent on how well satellites provide global coverage. Here the authors show a pair of four-satellite low-cost longer-life constellations that provide nearly continuous global coverage to support Earth observation.

## Introduction

Satellite services fundamentally shape telecommunication, navigation, and remote sensing services that are vital to the global economy. Sustained space-based Earth observation is critical for understanding and addressing global scale challenges, such as poverty, urbanization, water security, climate change, and epidemiological risks, to human health^1–4^. Despite the intrinsic value of sustaining space-based satellite services, the National Research Council has repeatedly warned that critical space infrastructures are at risk of collapse^4,5^. International coordination challenges^6^ combined with uncertain budgetary policies exacerbate the risk of reaching tipping points where the loss of space infrastructure could have long lived, if not irreversible impacts on critical data systems. Sustaining and advancing satellite constellations costs ~$10,000 per pound (0.45 kg) launched^7^ with total lifecycle mission costs often exceeding billions of dollars^8^. A core goal of this study is to improve the value proposition for those interests that cannot yet sustain their own global satellite services. A critical factor for addressing this challenge is reducing the total mass-to-orbit.

Propellant can account for a substantial portion of the mass budget of a mission, ranging from 6% (Global Positioning Systems) to 50% (the Clementine mission) of a satellite’s total mass^9^. Reducing the propellant required to perform a given mission reduces the cost-to-orbit, but also requires a detailed understanding of active mission control requirements, including active station-keeping, relocation, and deorbit. Active station-keeping refers to the ongoing control maneuvers to counter undesired changes in the constellation configuration due to perturbing accelerations (e.g., Earth’s geopotential, the gravity of the sun and moon, solar radiation pressure, etc.)^10^. However, with the right initial conditions, these forces can be harnessed to maintain, rather than disrupt, the configuration of a satellite constellation. This study presents the discovery of such designs via numerical optimization, nearly eliminating the propellant load required for a mission (see Supplementary Tables 1 and 2).

No mechanism presently exists to affordably resupply satellites with propellant, and so the quantity of propellant placed onboard prior to launch limits the lifetime of these vehicles. Active station-keeping propellant requirements thus impact both the design life and the launch mass of the vehicle. Classical benchmark designs, such as the Draim or Walker constellations, are theoretically known to attain ideal continuous global coverage^11,12^. However, in practice these designs pose severe tradeoffs between the need for more station-keeping propellant and the need for more spacecraft^13^. Regarding the Draim global coverage constellation^11^, prior studies have noted potentially high station-keeping costs^13^.

Here, we report four-satellite constellations with 24- and 48-h orbital periods that approach nearly continuous global coverage while substantially reducing mass-to-orbit and active station-keeping requirements (see Supplementary Figs. 1 and 2). These constellations represent a promising breakthrough for long life, low-cost global observation that could be of value in a broad range of scientific and commercial application areas.

## Results

### Nearly continuous global coverage

**Fig. 1:**
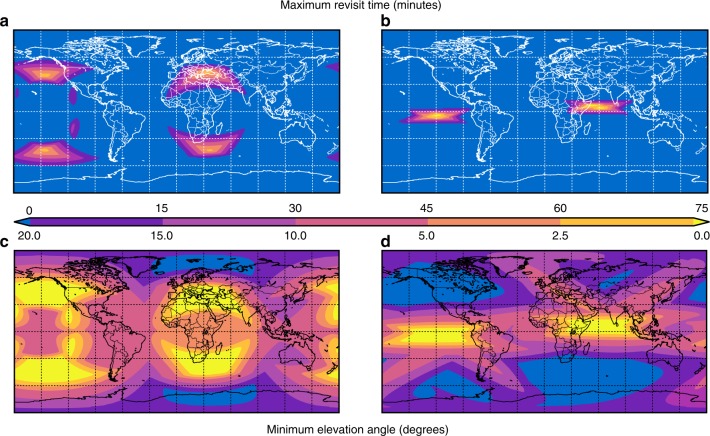
Maximum revisit time and minimum elevation angle. **a** Maximum revisit time for the 24-h constellation. **b** Maximum revisit time for the 48-h constellation. **c** Minimum elevation angle for the 24-h constellation. **d** Minimum elevation angle for the 48-h constellation. The top color scale represents maximum revisit time from 0 to 75 min. The bottom color scale represents minimum elevation angle from 0 to $2{0}^{\circ }$. The $x$ and $y$ axes are longitude and latitude, respectively.

The maximum revisit time for a point on the Earth represents the maximum time between accesses of that point by any satellite in a constellation. It is an important measure of the worst-case frequency of having geometric access to observe points on Earth. Figure 1a, b presents global contours of maximum revisit time for the discovered 24- and 48-h period constellations, respectively. The data contours account for station-keeping and drift over a period of slightly more than 8 years (3,000 days), with statistics calculated over a grid of ground target points with 1° spacing in latitude and longitude. The contour data indicate that 24- and 48-h-period constellations achieve continuous coverage over 86 and 95% of the globe, respectively. For those regions that experience outages, the maximum duration is on the order of 80 min once per day. The Supplemental Methods section provides additional background on the coverage metrics used in this study.

Orbit phasing describes both the positions of individual spacecraft relative to one another, as well as the position of the spacecraft relative to the rotating Earth. The specific phasing of the constellation determines the longitudinal position of regions that experience outages (Fig. 1a, b). Thus, these outage regions can be placed over regions of less interest to the constellation operator. An operator of a global agricultural monitoring constellation might, for example, position the coverage gaps over open ocean areas. Furthermore, these outage regions can be easily modified during the lifetime of the constellation if the regions of interest change. Small true anomaly phase changes to the constellation will shift these regions longitudinally for very little propellant cost, and can be performed multiple times.

The minimum elevation angle is defined as the minimum allowable angle between the horizon and any viewable spacecraft at a ground point. The intent of this angle is to account for terrain obstructions, such as buildings and mountains. If the Earth were a perfect sphere, without any features, then a minimum elevation angle of ${0}^{\circ }$ would be sufficient (ignoring atmospheric effects). However, these assumptions do not hold, and thus it is common in constellation analysis to use a positive minimum elevation angle to account for most types of terrain. In practice, it is common to require minimum elevation angles of 5–10°^14^. This is sufficient for most ground points, but is not applicable in all cases: consider an observer in a city with tall skyscrapers nearby, or an observer at the base of Mount Everest. The observed minimum elevation angle provides another measure of the quality of coverage for the constellations.

Figure 1c, d shows the minimum elevation angle observed across the 3,000 day simulation of the 24- and 48-h constellations. For the 48-h constellation, most of the Earth has a satellite at an elevation angle >${5}^{\circ }$ at all times; only the outage regions experience lower elevation angles. A vehicle at higher altitude has geometric access to a larger portion of the globe than the equivalent vehicle at lower altitude. One can see this demonstrated in the context of a commercial airline flight: at take-off one can see far less of the surrounding area than when at cruise altitude. This geometric effect extends to elevation angle as well: a satellite at higher altitude has, at any acceptable elevation angle, a larger field of view than a satellite at lower altitude. Thus, the lower altitude 24-h constellation (Fig. 1c) has the larger areas that experience elevation angles <${5}^{\circ }$.

### Minimizing propellant demands

Beyond initially attaining high-quality geometric coverage, another key aspect of a constellation design is the propellant required to actively manage the component satellites’ orbital configurations to maintain desired levels of coverage. The amount of propellant required is a function of the size of the maneuver(s) and the efficiency of the thrusters. The size of an orbit change maneuver is measured by the change in the velocity vector across the maneuver, generally referred to as the delta velocity ($\Delta {{V}}$). The thruster efficiency is characterized by a parameter called the thruster specific impulse. Given these two parameters, the propellant required for a maneuver or set of maneuvers can be computed using the rocket equation. In the field of orbit constellation design, it is common to use the $\Delta {{V}}$ as a surrogate measure of the propellant required^15^. The reason for this is that there are many different spacecraft thrusters available, each of which has a different efficiency. The constellation design is generally performed far in advance of spacecraft construction or thruster selection. Therefore, the thruster efficiency is generally unknown at the time of the constellation design. The $\Delta {{V}}$ measure can be easily translated into propellant requirements once the thruster efficiency is known. Minimizing the need for active station-keeping ($\Delta {{V}}$) by leveraging the energy from orbit perturbations implicitly mitigates the impact of thruster considerations on the constellation design by reducing the fraction of the vehicle mass that must be devoted to propellant. The Supplementary Methods section describes the station-keeping framework used in this study in more detail.

**Fig. 2:**
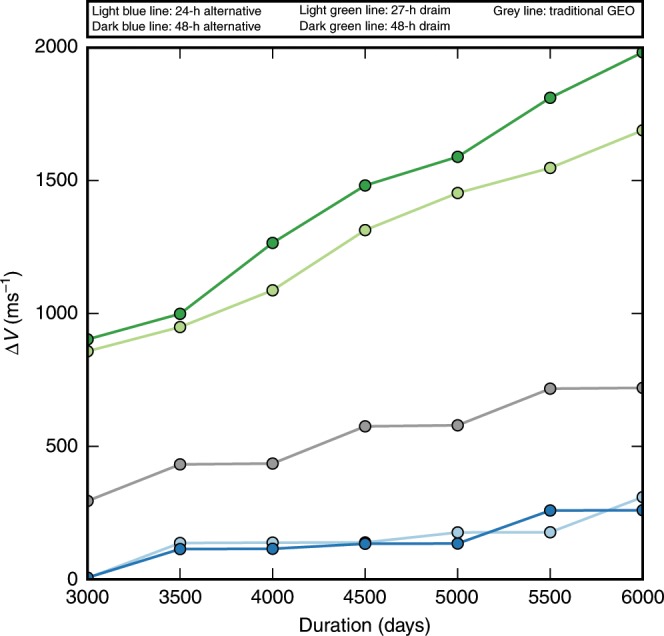
Maximum single-satellite station-keeping $\Delta {V}$ requirement. Mission duration varies from 3000 to 6000 days to capture a broad cross section of typical geostationary satellite lifetimes. Both constellations (light and dark blue circles) perform better than the GEO (gray circles) or benchmark Draim configurations (light green and dark green circles). While both 24- and 48-h constellations outperform the GEO configuration in terms of station-keeping propellant requirements by ~60%, the 24-h constellation retains the same orbit altitude, permitting similar conditions for payloads.

Figure 2 plots the maximum single-satellite $\Delta {V}$ station-keeping requirement as a function of time for the 24- and 48-h constellations, as well as for the 27- and 48-h Draim constellations and the widely employed geostationary equatorial orbit (GEO). A $2.{5}^{\circ }$ inclination control threshold is used. The constellations substantially outperform the Draim and GEO configurations in $\Delta {V}$ performance at all durations considered. The required station-keeping propellant is reduced in both cases by ~60%. To provide a sense of the different propellant requirements, a specific illustrative example is provided in Table 1, which lists a sample propellant budget for a notional spacecraft. These data demonstrate the impact of using the constellation configurations reported here rather than the equivalent GEO or Draim configurations on the mass budget for a vehicle. The constellations reduce the overall vehicle mass budget by ~60–65% for either chemical or electric propulsion systems. By harnessing the energy from the perturbing forces, the constellations considerably reduce the propellant required to operate in these orbit regimes. By reducing the example constellation’s propellant mass (Table 1) without adversely impacting its design life, this constellation design can potentially realize major mission-level benefits. This example reflects a 1,000-kg vehicle over a 6,000-day lifetime ($2.{5}^{\circ }$ inclination tolerance, ${5}^{\circ }$ argument of perigee tolerance), where values in parentheses represent the percent reduction in propellant mass for the 24- and 48-h constellations relative to GEO. Table 1 shows that the reduction in propellant mass is large enough to potentially accommodate another instrument, include another mission focus, double the design life, or some combination of these mission-level benefits. The reported constellation designs thus represent an alternative for propellant-averse missions, which can tolerate space vehicle motion relative to the ground which is fixed in a geostationary orbit.

**Table 1:** Comparison of propellant requirements.

Performance metric	GEO	27-h Draim	48-h Draim	24 h	48 h
$\Delta{{V}}$, m s^−1^	833	1689	2010	309	260
Mass, kg (Chemical $-{I}_{{\rm{sp}}}=230$ s)	308.8	527.1	589.8	128.0 (58.5%)	108.9 (64.7%)
Mass, kg (Electric $-{I}_{{\rm{sp}}}=1500$ s)	55.1	108.5	127.7	20.8 (62.2%)	17.5 (68.2%)

GEO geostationary Earth orbit, ${I}_{{\rm{sp}}}$ specific impulse of fuel type

### Using perturbations to sustain coverage

The individual spacecraft in constellations often occupy different orbital planes. The location of these planes relative to each other is specified by the right ascension of the ascending node (RAAN). Maintaining this relative spacing allows the constellation coverage to remain at nominal performance levels. There are many inertial configurations of a constellation that result in the same relative positions. Consider the simple example of three spacecraft whose RAANs are 0, 120, and $24{0}^{\circ }$; the relative planar spacing is $12{0}^{\circ }$. A constellation also might have RAAN values of 30, 150, and $27{0}^{\circ }$; these inertial values are different, but the relative spacing is the same. There is a subtlety regarding the RAAN: because the spacecraft planes are different, they will each experience slight differences in perturbative effects (e.g., nonlinear forces due to the gravity of the sun and the moon). Therefore it is good practice to ensure that these perturbative effects do not adversely affect the constellation, regardless of what the inertial RAANs might be. The Supplementary Methods section details the specific perturbations that are considered in this analysis.

**Fig. 3:**
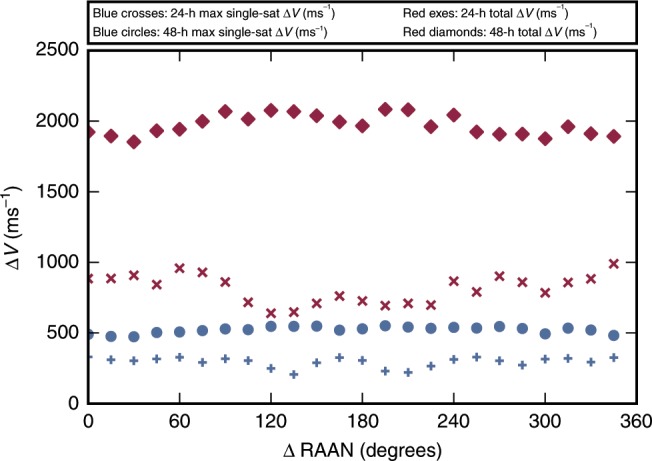
Station-keeping $\Delta {V}$ requirement. Total constellation (red) and maximum single satellite (blue) reported as a function of constellation and shift in RAAN applied uniformly to all satellites. The relatively flat curves indicate that constellations are not constrained to the reported gap locations, and can be adjusted with a limited impact to lifetime propellant needs.

The station-keeping performance of the 24- and 48-h-period constellations is fairly independent of the position of the constellation in RAAN. To demonstrate, Fig. 3 plots the total and maximum single-satellite station-keeping $\Delta {V}$ for both constellations as a function of a shift in inertial RAAN position while maintaining relative RAAN spacing. The reported data consider a mission duration of 6,000 days and an inclination control threshold of $0.{1}^{\circ }$, providing an aggressive station-keeping environment for study. The standard deviation of the maximum single-satellite $\Delta {V}$ for the 24- and 48-h cases is 35 and 23 m ${{\rm{s}}}^{-1}$, respectively. This demonstrates that the identified coverage gap locations from Fig. 1 can be shifted in epoch (or equivalently longitude) without dramatically impacting active station-keeping requirements. Constellation designers can thus select and control the location of the coverage gaps, providing crucial flexibility to how the reported designs can be used for specific missions.

**Fig. 4:**
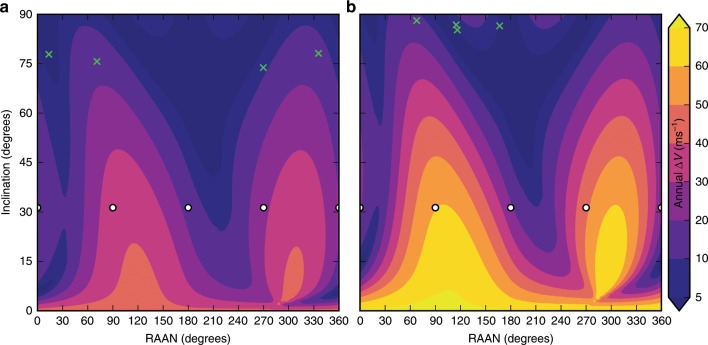
Annual station-keeping $\Delta {V}$ required to maintain inclination. Varies according to inclination and right ascension averaged over 10 years. **a** Contours for 24-h orbits. **b** Contours for 48-h orbits. The green crosses on each plot indicate the position of the satellites in the constellations, and white circles indicate the location of satellites in a Draim constellation.

Figure 4a, b plots the position of the satellites in each constellation on annual station-keeping requirement contours in RAAN and inclination space for 24- and 48-h orbit periods, respectively. The contours in the plot show the $\Delta {V}$ required to maintain a given inclination using semiannual station-keeping maneuvers. The shape of the contours results primarily from third-body interactions with the sun and the moon. Effectively, dark purple regions on the map show where satellites can harness the energy of these perturbations to maintain configuration rather than struggle against them. Both identified constellations, identified by green crosses lie in these advantageous regions of the space. In addition, the constellations lie in inclination regions with relatively flat contouring in the direction of RAAN. This contrasts with the contouring at the Draim inclination of $31.{3}^{\circ }$, indicated by white circles, which at both orbit periods passes through regions with relatively high annual station-keeping requirements. By positioning satellites in inclinations with more benign variations in station-keeping requirements across RAAN, the satellites do not experience significant coverage-degrading differential motion between one another. This allows the satellites to forego RAAN station-keeping without adversely affecting the constellation’s coverage performance. The constellations reported here represent only two of many designs identified during the search process. Details regarding the broader results of the search are available in Supplementary Figs. 1 and 2. The orbital elements for the reported constellations are shown in Supplementary Tables 1 and 2.

## Discussion

Throughout the 1970’s, the minimum number of spacecraft required to realize total continuous coverage of the Earth surface was thought to be five^12^. Then, Draim published his famous result in 1986 that only four spacecraft were required^11^. These findings were based on a low-fidelity modeling of spacecraft motion. Unfortunately, subsequent researchers noted that when higher fidelity perturbations are enabled in the simulation of the Draim orbits, the propellant required to maintain these orbits can be significant^13^. Thus, while this solution was academically interesting, practical implementation proved to be overly difficult.

This paper reports a pair of four-satellite constellations with common period and similar inclination between satellites that very nearly realize the elusive goal of continuous global coverage. The price of a slight degradation in their coverage has bought a significant reward: operational feasibility. Moreover, the modest gaps in coverage can be flexibly moved to longitudes of least importance at minimal costs to missions. By including the perturbative effects of the sun, the moon, and the asymmetric Earth in the analysis, constellations have been discovered that harness these forces and use them for positive gains (i.e., reduced costs and longer design life). The resulting orbits require dramatically less station-keeping propellant than the Draim constellation and other commonly used constellations, permitting reduced costs and longer potential design lives. In this way, the orbits reduce the barrier of entry for new nation-state and commercial members of the growing space community to own and operate constellations that provide nearly continuous global coverage, enabling these interests to independently sustain global satellite services.

The constellation designs reported here account for many considerations made in early mission planning. However, they are likely not optimized for specific mission objectives, which would likely require minor modifications, yet are worthy of further consideration by mission planners. Not all missions will find added utility with this class of constellation design compared with other design options, such as medium-earth orbit, geostationary orbit, or highly elliptical orbits (HEO).

Geostationary orbits in particular afford mission planners a fixed subsatellite point that may present an advantage for broader end-to-end mission considerations over the reduction in station-keeping propellant reported here for the designs. However, many governments and commercial interests appear to be pivoting their GEO-based capabilities to low earth orbit. As a result, significant investments are being made to reduce the regrets associated with a moving subsatellite point, which may increase the benefits for the reported designs in this study. The reported designs provide nearly continuous coverage, with repeatable gaps as reported here. However, GEO constellations do not provide any access to the planet’s polar regions, which is also a driving requirement for some mission areas. Some traditional GEO constellation designs which serve missions that have polar coverage requirements augment that baseline constellation with services from HEO platforms to fill the gap. Because the gaps in coverage experienced by the reported designs are fixed on the ground, it is possible that for missions that require truly continuous global coverage that the reported designs may likewise be augmented by partnering with or hosting mission payloads on some of the many available GEO space platforms. In these circumstances, the reported constellation designs represent a foundation for broader end-to-end architecture, particularly for disadvantaged entrants who may be able to cover gaps in their capability through partnerships and hosting arrangements.

The designs reported here likewise account for some early considerations in constellation operation and management, but do not represent a fully optimized constellation management approach for an operational system. Additional reduction in station-keeping requirements may be realized through alternate mission management approaches and by providing the optimizer with control over decisions related to that in identifying constellation design alternatives.

## Methods

### Many objective evolutionary optimization

**Fig. 5:**
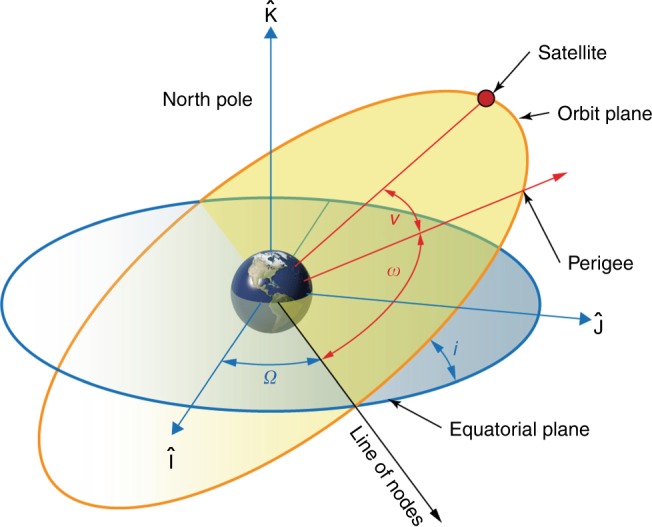
Angular orbital elements referred to in this study. Inclination ($i$) is the angle at which the orbit is tilted out of the equatorial plane. Right ascension of the ascending node (${\Omega }$, RAAN) is the angle of rotation around the Earth spin axis referenced by convention to the direction of the sun at the vernal equinox. The argument of perigee ($\omega$) describes the position of the perigee point relative to the point on the orbit which ascends across the equator. True anomaly ($\nu$) is the angle in the orbit plane between a satellite at an instant in time and perigee. In addition to these angular elements, the semimajor axis ($a$) and eccentricity ($e$) describe the size and shape of the orbit, respectively.

The 24- and 48-h-period constellations presented in this study were discovered using multiobjective evolutionary optimization techniques to intelligently search the orbital design space. Figure 5 provides an illustration of orbital elements that must be defined for each of the satellites in the 24- and 48-h-period constellations. These elements define the dynamics and coverage performance. The simulation–optimization framework used in this study was implemented on the University of Illinois Blue Waters petascale supercomputer consisting of hundreds of thousands of cores. The Borg multiobjective evolutionary algorithm (MOEA) was selected as the optimization tool for this work due to its efficient design for massively parallel search and its ability to perform robustly for severely challenging nonlinear problems^16^. The results reported here were attained from search evaluations of over five million simulated orbital designs. As summarized below, high-fidelity orbit propagation and careful simulation of station-keeping significantly increased the computational demands of simulating each candidate design. More details on the simulation–optimization framework can be found in ref. ^17^.

### High-fidelity orbit propagation and station-keeping

Modeling the orbital motion of the satellites in high fidelity allows the Borg MOEA to identify solutions that maximally harness the energy of naturally occurring perturbation forces to improve global coverage while simultaneously simulating station-keeping requirements. The Aerospace Corporation’s SHARK propagator accomplishes long-term orbit propagation in this work. SHARK is a general-purpose and configurable orbit propagator that can numerically simulate the motion of any Earth-orbiting spacecraft using models that have no known numerical singularities. SHARK is configured in this study to include a 4 × 4 representation of Earth’s gravity as well as solar and lunar third-body gravitational perturbations; these were selected to maintain consistency with prior results^13^. Other perturbing effects, such as drag, solar radiation pressure, and higher order geopotential terms, are generally dominated by those modeled in this study at the orbit periods of interest. Supplementary Fig. 3 shows that, although higher order geopotential terms could be incorporated in this framework, their inclusion does not materially affect the results. The model simulates station-keeping maneuvers to maintain the radius of perigee, inclination, and relative phasing between constellation members. The radius of perigee is maintained to within 1,000 km and inclination is maintained to within $2.{5}^{\circ }$ of the initial configuration. Relative phase positioning between satellites is maintained with a feed-forward active control that operates on a 2-week cycle. In general, the dominant source of station-keeping $\Delta {V}$ in the orbits of interest is inclination control. This fact known a priori about the design space motivated the decision not to incorporate RAAN station-keeping. A series of Chebyshev polynomials capture the final numerically generated orbit state history for each satellite in memory, which permits efficient evaluation of constellation coverage performance. The Supplementary Methods section provides additional details on all of the simulation and search components of this work.

### Problem formulations

The MOEA used high fidelity simulation to search for orbital configurations that balance conflicting objectives. These include minimizing the global maximum revisit time, minimizing the 95th percentile global revisit time, minimizing the global average response time, and minimizing the maximum station-keeping $\Delta {V}$ for any satellite in the constellation over 3,000 days of orbit propagation. The global maximum revisit time represents the maximum gap in coverage for any single ground location on the Earth by at least one satellite. The 95th percentile global revisit time provides a measure of the gaps in coverage that are not skewed by significant outliers. Global average response time is a measure of the average wait from any random point in time to have access to any target ground location on Earth. These objectives provide a feedback to the MOEA for identifying constellations which approach continuous global coverage. The final objective to minimize the maximum station-keeping $\Delta {V}$ required by any satellite in the constellation drives the optimization to solutions with lower orbit maintenance requirements and enables the MOEA to trade coverage for reduced $\Delta {V}$. The maximum single-satellite $\Delta {V}$ is minimized rather than total satellite $\Delta {V}$ to encourage an equal distribution of maintenance among all members of candidate satellite constellations, which is a common requirement in operational design applications. The Supplementary Methods section provides additional detail regarding the search domain explored by the MOEA for the 24- and 48-h formulations, including visualization of the orbit search domain in Supplementary Fig. 4 and description of the constellation search domain in Supplementary Tables 3–5.

The approach used to discover the reported constellation designs represents just one path to accomplishing this work. Alternative formulations of the problem can arrive at comparable designs or potentially even designs which achieve marginally superior performance. The exploration of refined problem formulation for improved outcomes is a subject of future work.

## Supplementary information

Supplementary Information

## Data Availability

The authors declare that the data supporting the findings of this study are available within the paper and its Supplementary Information.

## Code availability

The code used to evaluate the constellation designs presented in this study exploit standard numerical techniques for astrodynamics simulations and coverage performance analyses that have a detailed publication history. The Supplementary Information provides readers with a summary of the numerical solution methods used in our analysis as well as specific orbital elements so that the interested reader will be able to replicate or extend our reported results.
