# Supplementary material for: Low cost satellite constellations for nearly continuous global coverage
Source: Nat Commun. 2020 Jan 10;11:200. doi: 10.1038/s41467-019-13865-0 (PMC6954248; doi:10.1038/s41467-019-13865-0)
Supplement: Supplementary file 1 — Supplementary Information [file 41467_2019_13865_MOESM1_ESM.pdf]

Supplementary Information: Low Cost Satellite Constellations for Nearly Continuous Global Coverage

Lake A. Singh[1], William R. Whittecar[1], Marc D. DiPrinzio[2], Jonathan D. Herman[3], Matthew P. Ferringer[4] & Patrick M. Reed[5]

[1]*Performance Modeling and Analysis Department, The Aerospace Corporation, Chantilly, VA 20151*

[2]*Mission Analysis and Operations Department, The Aerospace Corporation, Chantilly, VA 20151*

[3]*Civil and Environmental Engineering, University of California, Davis, CA 95616*

[4]*GEOINT Innovations Office, The Aerospace Corporation, Chantilly, VA 20151*

[5]*Civil and Environmental Engineering, Cornell University, Ithaca, NY 14853*

1 Supplementary Discussion

For years it was thought that the minimum number of satellites to provide global coverage was five. Draim [1] showed that although global Earth coverage cannot be provided with four satellites in circular orbits, it can be achieved with a novel design consisting of three elliptic inclined satellites and one circular equatorial satellite having one-half the period of the rest of the constellation. Draim [2] refined this result with a more elegant four-satellite constellation design that offers continuous global coverage using four elliptical orbits with common period and inclination. Unfortunately, Chao [3] discovered that the Draim orbits degrade in their performance under the effects of luni-solar gravitational perturbations. Chao went on to describe the degradations in ground coverage:

16% for the 27-hour constellation, and 32% for the 48-hour constellation, both over a 3,000-day lifetime. Chao also explored orbit maintenance to maintain the configuration of the constellation, and found that the vehicles in the constellation would need to expend as much as 830 m/s for the 27-hr orbit and 835 m/s for the 48-hr orbit to control the inclination and argument of perigee in order to maintain 100% continuous coverage for 10 years. The 1990 work by Chao is the motivation of this study.

Chao's work acknowledges that one way to reduce the station-keeping requirements he identified is to bias the initial orbital elements. If all of the orbits comprising the constellation begin at their nominal values, then perturbations will most likely induce drift away from these nominal values at the constellation level. If biased correctly, the orbital elements can be configured such that they drift through their nominal values over the life of the constellation. The biased initial conditions can allow the mean value of the orbital element to be centered on the nominal value, which can help minimize overall displacement of the orbital elements and the resulting impact on constellation performance.

Armed with the knowledge that continuous global Earth coverage cannot be provided with four circular satellites [1], and that the four-satellite elliptical constellation described by Draim is significantly degraded by perturbations [3], we sought to answer two questions. (1) Is it possible to find a four-satellite constellation that significantly reduces the orbit maintenance required by the Draim constellation, while seeking at least near global coverage? (2) What is the trade-off between the station-keeping costs and the amount of global coverage degradation? Use of con-

sistent modeling assumptions and fidelity facilitated comparison with Chaos benchmarking and is shown in Section 2.2 of the Supplement to provide sufficient numerical accuracy to support early architecture design efforts. The work performed in pursuit of exploring these questions led to the constellation designs reported in this study, which represent an unreported region of the broader trade-space of constellation design alternatives for near global coverage.

Supplementary Tables 1 and 2 detail the orbital elements for the 48- and 24-hour period constellations reported in this study, respectively. The orbital elements reported here are Brouwer mean elements in a true-of-date frame. The epoch for the elements as presented is November 26, 1995 at midnight GMT in order to maintain consistency with prior published benchmark results [4]. Although the reported designs represent only two of the many tradeoff alternatives identified in this study, they represent some of the most significant findings given their balance of nearly continuous global coverage and greatly reduced station-keeping propellant costs.

The constellation designs vary significantly from the classical benchmark Draim constellation [2]. One such significant difference is the eccentricity (e) of the reported orbits. Eccentricity describes the shape of the orbit. Satellites in orbit around a planet have eccentricity between 0 and 1; a value of 0 means the orbit is perfectly circular, and larger values mean the orbit is more elliptical in shape. The low eccentricity values of the orbits indicate that they are nearly circular for both the 24- and 48-hour period constellations, in contrast with the eccentricity of 0.263 for a Draim constellation.

As an orbit becomes more eccentric, the point of closest approach to the Earth (perigee)

Supplementary Table 1: Brouwer mean orbital elements for one 48-hour constellation identified by the evolutionary algorithm.

Element	Sat 1	Sat 2	Sat 3	Sat 4
a (km)	66,931.2	66,931.2	66,931.2	66,931.2
e	0.030	0.018	0.018	0.018
i (deg)	86.49	86.81	88.12	85.26
Ω (deg)	166.79	114.81	67.67	116.19
ω (deg)	134.31	153.05	148.86	155.39
ν_0 (deg)	180.00	36.82	175.18	306.35

Supplementary Table 2: Brouwer mean orbital elements for one 24-hour constellation identified by the evolutionary algorithm.

Element	Sat 1	Sat 2	Sat 3	Sat 4
a (km)	42,163.6	42,163.6	42,163.6	42,163.6
e	0.020	0.010	0.021	0.018
i (deg)	75.71	73.83	77.86	78.15
Ω (deg)	71.03	269.68	13.57	335.59
ω (deg)	65.75	359.72	169.28	258.15
ν_0 (deg)	180.00	325.35	231.50	271.44

moves closer to the Earth, while the farthest point (apogee) moves farther away. In most cases, it is important to control the orientation of the perigee point. The argument of perigee (ω) describes the position of the perigee point relative to the point on the orbit which ascends across the equator, as illustrated in Figure 5. Orbits with low eccentricity require less propellant to maintain the argument of perigee than orbits with larger eccentricity. Because the constellations have small eccentricities, they essentially eliminate the need for argument of perigee maintenance through propulsive maneuvers.

The orientation of an orbit in space can be described by a series of Euler angle rotations; two of these angles are called the inclination (i) and the right ascension of the ascending node (RAAN). The inclination is the angle at which the orbit is tilted out of the equatorial plane. Once the orbit is tilted out of the equatorial plane, it can be rotated around the Earth spin axis to define any one of a continuum of orbit planes. The RAAN is this angle of rotation around the Earth spin axis referenced by convention to the direction of the sun at the vernal equinox. The classical Draim constellation configuration places satellites in equally-spaced planes, such that the difference in the RAANs from one spacecraft to the next is always $90°$. However, the constellations proposed here do not share this uniform placement in RAAN.

In addition to the difference in RAAN spacing, the orbits exhibit much higher, nearly polar inclinations when compared with the Draim inclination of $31.3°$. To understand the advantage of these higher inclinations, it is necessary to understand a fundamental perturbation to orbital motion. Anisotropies in the shape and mass distribution of the Earth create spatially-variant perturbations

on the gravitational force experienced by a spacecraft. These perturbations cause the RAAN of orbits to change over time at a rate which is a function of the orbital parameters. The perturbations have a non-uniform effect on orbits with inclinations similar to the Draim inclination, causing the orbit planes to shift relative to one another over the course of the lifetime of the constellation. However, at nearly polar inclinations the perturbations cause a smaller and more uniform motion of the orbit planes. This reduces the station-keeping maintenance required to maintain the shape of the constellation.

The constellation designs reported here represent just two of the most attractive designs identified by the MOEA during its exploration of the feasible design space. Supplementary Figure 1a illustrates the efficient frontier of constellation designs discovered throughout this work. The MOEA identifies families of constellation designs with varying levels of compromise between the competing objectives of maximizing coverage performance and minimizing station-keeping requirements. Circles represent 48-hour designs, and diamonds represent 24-hour designs identified by the MOEA. The efficient frontier of constellation designs divides into two families. In the center of Supplementary Figure 1a lies a family of 24-hour constellation designs which have inferior coverage performance, but offers a marginal improvement of less than 1 m/s of station-keeping performance when compared with the family at the bottom-left of the figure. A 1 m/s reduction in station-keeping requirements is negligible in the context of a spacecraft propulsion system. Thus, these designs are excluded from additional consideration because this tradeoff is not considered attractive from a mission perspective.

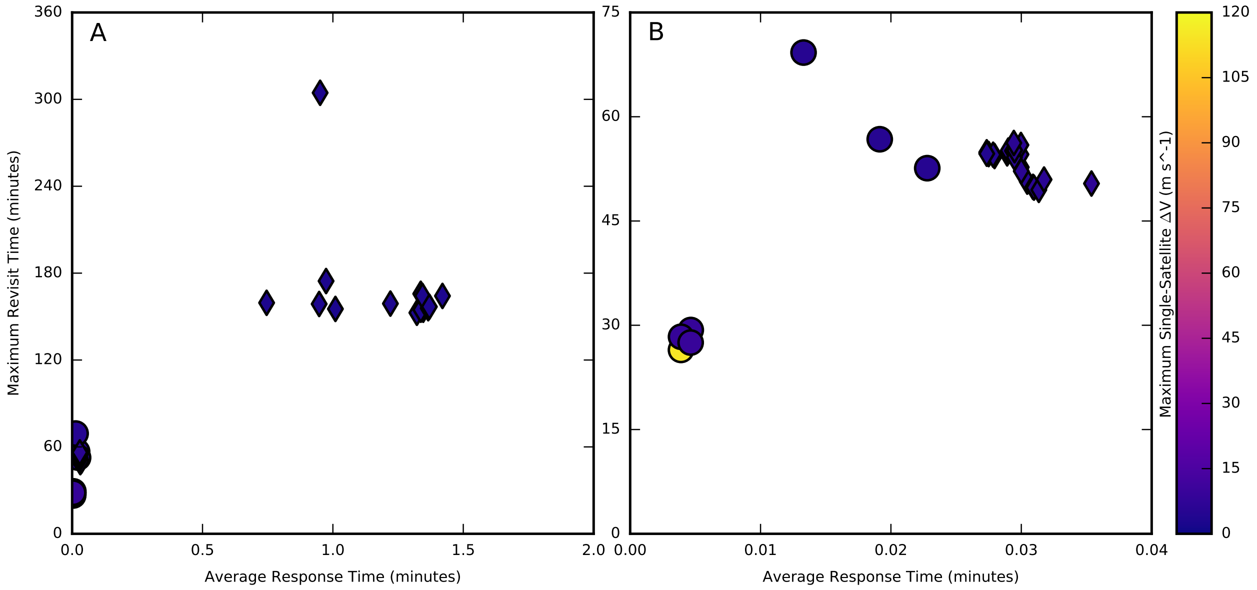

Supplementary Figure 1: Non-dominated constellation designs. (A) Full range of tradeoffs for Maximum Revisit Time (0-360 minutes) and Average Response Time (0-2 minutes). (B) Zoomed view of the highest performing designs for Maximum Revisit Time (0-75 minutes) and Average Response Time (0-0.4 minutes). All constellations were identified by the MOEA during its exploration of the design space. Color indicates the maximum single-satellite station-keeping required over approximately eight years of operation. Circles and diamonds represent 48-hour and 24-hour designs, respectively.

Supplementary Figure 1b shows the family of constellation designs at the bottom-left of Supplementary Figure 1a in additional detail. The closer view reveals that, as with the 24-hour designs, the 48-hour designs consist of two distinct families which trade in coverage performance and station-keeping performance. One family maximizes coverage performance at the cost of station-keeping requirements, and the other minimizes station-keeping requirements at the cost of coverage performance. Of the 48-hour designs which optimize coverage performance over station-keeping performance, one design takes this trade to the extreme. This option at the bottom-left of the population requires up to 114 m/s of station-keeping per satellite over 8 years. Although this option represents the best coverage performance identified by the MOEA, accepting a marginal reduction in coverage affords a significant improvement in station-keeping performance. This illustrates the contention between these two areas of performance, and implies the existence of a spectrum of design options which offer designers varying levels of compromise. The 24-hour family of designs lies behind the 48-hour families in the coverage performance space, indicating a trade between coverage and orbital period. The reason for this trade mirrors the logic provided in the main paper for the difference in minimum elevation angle performance: a vehicle at a higher altitude can see more of the Earth under it than the same vehicle at a lower altitude. The additional coverage provided by each vehicle at a higher altitude drives a corresponding improvement in the constellation-level coverage performance evaluated in this study.

Supplementary Figure 2 shows a reduced population of constellation designs attained by applying preferences to the efficient frontier and reducing the objective space. Translucent options are those which have high station-keeping requirements, poor coverage performance, or are not on

the efficient frontier in the reduced objective space attained by neglecting the 95th percentile global revisit time objective. Of the resulting population of constellation designs, additional analysis and comparison of coverage visuals like those shown in Supplementary Figure 1 lead to the selection of the reported constellations as annotated in Supplementary Figure 2. The broader search results thus contain constellation designs in addition to those reported here which may be attractive to constellation designers with alternative preferences.

Even the broader results reported here represent only a narrow cross-section of possible designs for simultaneously attaining high coverage performance and low station-keeping requirements. The Borg MOEA uses ϵ-dominance sorting to maintain a focus on exploration of the decision space for attractive design alternatives, rather than enumeration of all attractive alternatives which would be intractable even with petascale computing resources [5]. Although ϵ-dominance sorting enables efficient exploration of possible distinct alternatives, it also obscures a likely continuum of constellation designs offering varying levels of compromise in coverage, orbit period, and station-keeping requirements. The two specific constellation designs reported here thus represent the intersection of the investigators' preferences and the sampling of the possible decision space explored by the MOEA. The same framework for discovery of constellation designs employed to identify these designs can be used to find similar designs which better align with designers' specific application and preferences.

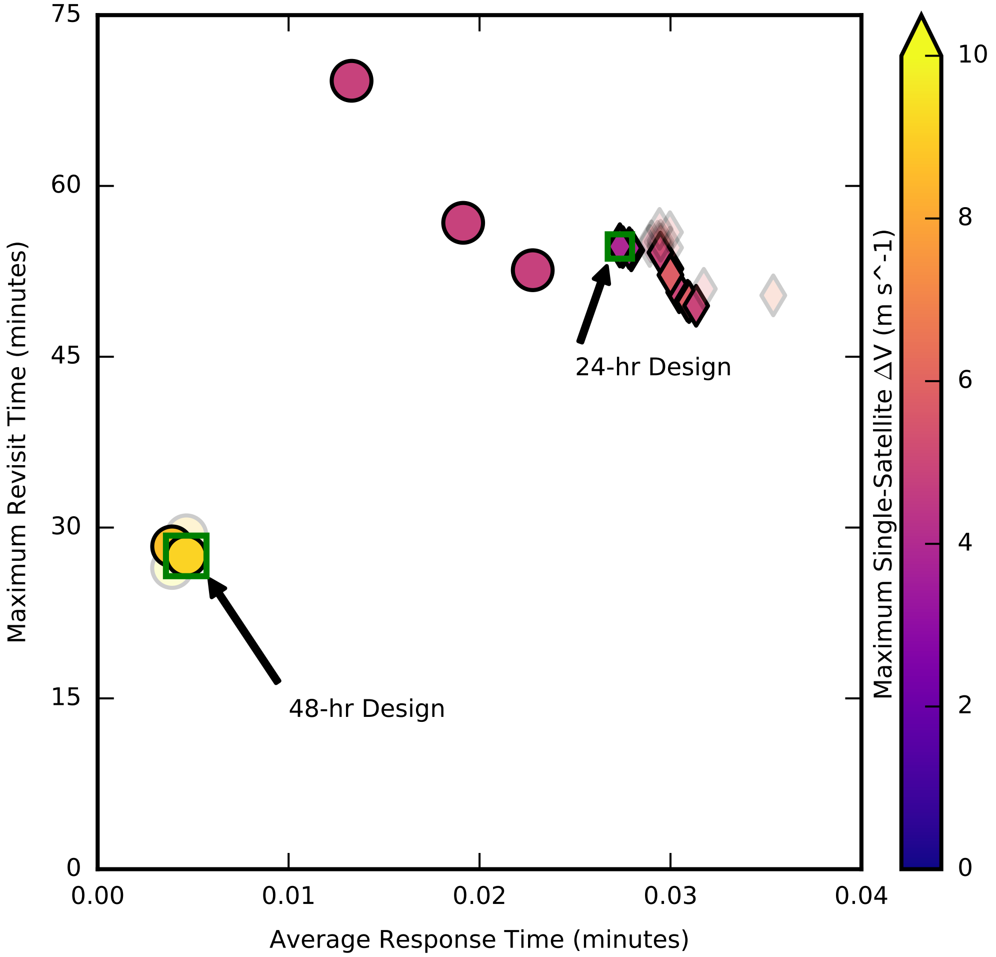

Supplementary Figure 2: Reduced population of designs after applying investigator preferences. Translucent options have high station-keeping requirements, poor coverage performance, or are not on the efficient frontier in the reduced objective space attained by neglecting the 95th percentile global revisit time objective. The annotated 48-hour and 24-hour designs are reported in the study. The presence of nearby options in the performance space highlights the diversity of constellation designs available to attain long-life constellations for nearly continuous global coverage.

2 Supplementary Methods

At the most general level, the performance of a constellation of satellites is assessed using metrics that quantify the specific performance characteristics that relate to the geometric access they provide for global observations. Geometric access represents time periods when a satellite in the constellation can perform its mission in sensing a specific location of interest. At its most general, access is calculated as time periods when any satellite within the constellation has an unobstructed line of sight to a target. When a mission is more defined, the locus of targets a satellite has access to at any instant may be constrained by a number of factors which inhibit the satellites ability to perform its mission. These constraints take the form of availability and visibility constraints. Availability constraints limit the time during which a satellite is available to perform its mission, representing operational considerations such as duty cycle. Visibility constraints constrain the geometrical conditions between targets, satellites, and potentially other bodies relevant to performing a mission. Because of the general nature of this work, no availability or visibility constraints are levied in the calculation of access. However, the content in the Nearly Continuous Global Coverage section of Results in the main text studies the sensitivity of the reported constellation performance to the target elevation angle, which is the angle between the horizon and the satellite from the targets perspective. This is a commonly used visibility constraint to account for terrain masking on the ground when designing a constellation.

Constellations typically do not have continuous access or coverage to a target, so many access metrics measure the gaps in coverage. This work considers three gap-based access metrics:

maximum revisit time, 95th percentile revisit time, and average response time. The maximum revisit time is defined as the longest gap in coverage experienced by a target during the simulation period of the constellation. The 95th percentile revisit represents an amount of time longer than 95 percent of all gaps experienced by a target. Average response time represents the average amount of time remaining until the next access from any random point in time during the simulation. It is calculated as the sum of the squares of all of the gap times experienced by a target during the simulation duration divided by twice the simulation duration.

As mentioned previously, each of these metrics is evaluated between the satellite constellation and a single target. Organizations which choose to invest in a satellite constellation typically intend to use that constellation to perform a mission for large regions or even the entire earth, in which case evaluation at a single point provides an inadequate picture of performance. Constellation designers overcome this by aggregating access performance across a set of targets which spans the region of interest. The results presented in this work represent aggregated access performance from a target set which spans the globe. The highest value calculated for any target point across the globe represents the maximum and 95th percentile revisit metrics reported, whereas the average value from all points across the globe represents the average response time reported.

The lifetime simulations in this work employ the Aerospace Corporations SHARK propagator. SHARK is a general-purpose orbit propagator that can numerically simulate the motion of any Earth-orbiting spacecraft using models that have no known numerical singularities. Brouwer [6] and Chao [7] provide equations which approximate the secular motion that results from these pertur-

bations. However, over longer time frames solutions derived from these approximations can drift potentially far away from the true solution. Although this drift is acceptable for some applications, it can lead to significant accuracy issues in the evaluation of constellation coverage performance due to the sensitivity of this performance to constellation phasing. Key technical features for the SHARK propagator are summarized below for (1) the integrator and (2) quantifying perturbations.

The default integrator is the eighth order DOPRI8 Runge-Kutta integrator [8]. An alternate option for conservative systems (no drag, no SRP) is the Runge-Kutta-Nystrom method, which is the primary method employed in this work [9]. Both of these algorithms are desirable for their accuracy and speed. While higher order integrators require more steps, they likewise allow larger steps to be taken. This directly translates to higher computational efficiency and speed, as noted by Montenbruck [10]. The RKN12(10)17M integrator described by Dormand [9] permits faster computational analysis than standard Runge-Kutta techniques for conservative systems, which is consistent with the perturbations modeled in this work. Both methods use an adaptive step size technique [11] to integrate in nonsingular cartesian coordinates (position and velocity) or in nonsingular equinoctial elements.

To quantify perturbations, a full geopotential model is available, supporting field sizes up to 80x80. The standard Legendre expansion can be used (which has singularities at the poles). Alternatively, the user can select a non-singular technique described by Pines [12]. A variety of geopotential fields is supported, including EGM-96, EGM-2008, GGM02C, GGM03C, EIGEN-GLO4C/5C/6C, JGM3, GEM-T1, WGS-72, and WGS-84. Other fields can be loaded in a separate

file. Normalized and non-normalized coefficients are supported. Solar and lunar gravity perturbations, as well as solar radiation pressure perturbations, are also supported [13]. Atmospheric drag is supported via the ubiquitous ”cannonball” model. The user can select from a variety of atmospheric density models, including: the 1962 US Standard Atmosphere, the Harris-Priester density model, the Drag Thermosphere model, The 1994 Drag Thermosphere Model, MSIS-86, MSIS90, MSIS-2000, and Jacchia 1971. Nutation and precession modeling is supported via either the IAU-1980 model or the IERS 1996 model. Finally, the user can also enable solid earth tide perturbations.

The propagation assumptions used during optimization (main text methods subsection High-Fidelity Orbit Propagation and Station-Keeping) are sufficient to represent the orbital dynamics of the constellation while conserving wall-clock compute time. Supplementary Figure 3 illustrates the difference in maximum revisit time performance evaluated with the orbit propagation assumptions used in this work and more precise assumptions. Specifically, the difference contours show the impact of using a more detailed 36x36 EGM-96 Earth geopotential model and including precession and nutation terms over 4,000 days of propagation. These additional areas of fidelity contribute only minor changes in the long-term coverage performance reported in this work.

The propagator provides the orbit states necessary to assess coverage performance. Evaluation of the measures discussed in Section 2.1 rely on computing the start and stop times of the visibility of a given satellite to a specified ground point. This process is complicated by a numerically generated orbit state history, which can be solved in a brute force manner via repeated root finding around times where satisfaction of the constraints associated with visibility change.

A more computationally efficient approach to accomplishing this is to store the spacecraft state history in a set of orbit tables. The process of creating and using these orbit tables is as follows. As the spacecraft state is propagated forwards in time, the integrated states are stored over a prescribed interval of time. Once the end of this interval is reached, a set of Chebyshev polynomials are fit to the states over this time interval and then stored in memory. The integrated states are then discarded, and only the polynomial coefficients are retained. Repeating this process, the orbit table is built up over the entire propagation time of the orbit. Once the table is complete, the evaluation of the spacecraft state at any point in the propagation duration can be performed via a table lookup of the time index, and a set of polynomial evaluations. The full set of necessary coefficients needed to represent the longest propagation duration used in this work (6,000 days) for four vehicles can be stored in memory without requiring disk access. This approach sidesteps the need to perform any back propagation during the evaluation of access statistics, which permits significant acceleration of the evaluation over repeated numerical integration.

The station-keeping method used in the orbital simulations follows an anchor-chaser paradigm. Although the anchor spacecraft performs maneuvers to maintain certain orbital parameters, it does not actively seek to maintain any specific in-track phasing with other members of the constellation. Similarly, the chaser spacecraft performs maneuvers to maintain specific orbital parameters, but it also is required to maintain an in-track position relative to the anchor spacecraft. The anchor spacecraft maintains its semi-major axis, perigee radius, and inclination. These orbital elements are constrained within pre-specified lower and upper limits. In the simulations, this is implemented in a simple manner that controls each orbital parameter separately. There is no effort to combine the

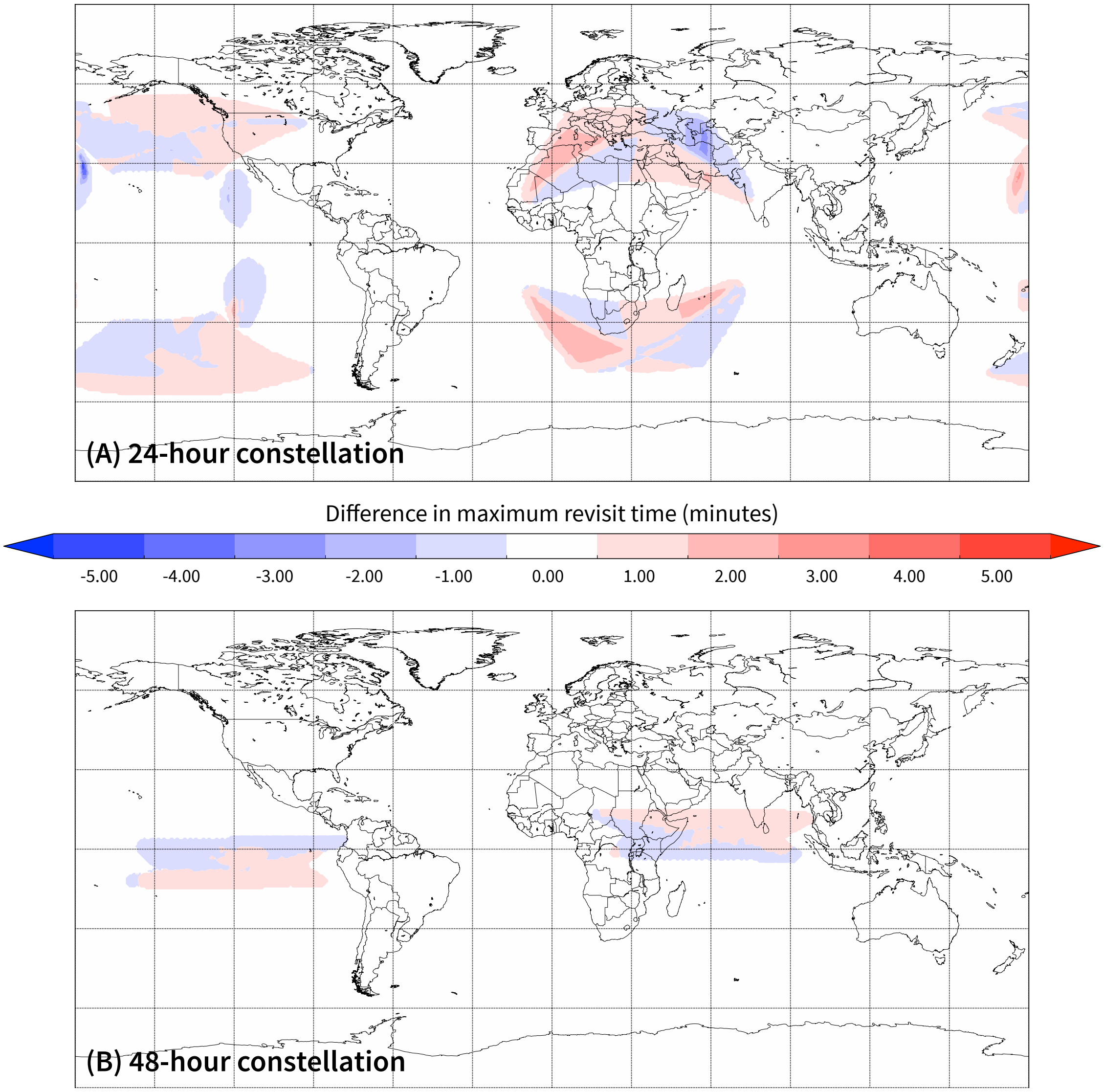

Supplementary Figure 3: Difference in maximum revisit time. (A) 24-hour and (B) 48-hour constellation design. Red areas represent regions where using a higher fidelity Earth geopotential model and including precession and nutation terms increases the maximum revisit time realized by the constellation designs over 4,000 days of propagation. Areas in blue represent the opposite. No area experiences a difference of more than 5 minutes for either constellation design. The x and y axes are longitude and latitude, respectively.

maneuvers to reduce the required ΔV. The following algorithm describes the method employed to identify maneuver times and magnitudes:

1. Propagate the spacecraft until one of the specified elements (semi-major axis, perigee radius, inclination) crosses a lower/upper boundary.
2. Compute the maneuver that will return the offending element to its nominal value.
3. Execute the maneuver at the specified location.
4. Continue with the propagation until the next violation.

The allowable ranges of the orbital elements of the lifetime simulation are shown in Supplementary Table 3. As an example, with a nominal initial inclination of 86.49°, the allowable range would be 83.99° to 88.99°. When the inclination exceeds either of these bounds, the model executes a maneuver to return the inclination to its nominal value of 86.49°.

There are three primary goals of maintaining these specific parameters (semi-major axis, perigee radius, and inclination): (1) By controlling the semi-major axis, the orbital period is maintained, (2) By controlling the perigee radius, the eccentricity is maintained, and (3) By maintaining all three of these parameters, a secondary effect is to approximately control the secular rate of the RAAN. Supplementary Equation 1 describes this rate as a function of the three controlled parameters, as well as the Earth's J_2 term, and the planet's radius, R_{e}. Controlling the three orbital parameters in this equation thus implicitly leads to control of the secular RAAN rate as well. For

the anchor spacecraft, there is no explicit effort to directly maintain the RAAN, the argument of perigee, or the longitude. It is possible that more sophisticated approaches to coordinated station-keeping of the constellation could lead to designs which further improve on the results presented here.

$$\dot{\Omega}_{\mathrm{sec}} = -\frac{3nR_{\mathrm{e}}^2 J_2}{2a(1-e^2)}\cos(i) \tag{1}$$

Regarding the three chaser spacecraft, they are required to maintain the perigee radius and inclination, as well as the in-track phase (position relative to the anchor). For the former two elements, the techniques used are the same as for the anchor. For in-track phasing, each chaser maintains a station-keeping cycle of 14 days. At the beginning of each cycle, the spacecraft performs a maneuver that targets a particular value of semi-major axis. This value is chosen such that after 14 days, the spacecraft will arrive at the nominal phase offset. During the 14-day cycle, the phase value drifts freely, but at the end of the cycle, the phase returns to the nominal value. The maximum phase excursions during the station-keeping cycle can be controlled by increasing or decreasing the station-keeping cycle duration, which will increase or decrease the excursions, respectively. For these constellations, the 14-day station-keeping cycle provides phase control within approximately 0.5°.

Controlling the semi-major axis indirectly maintains the phase of the chasers. Thus, the chasers also maintain control of the three key parameters: semi-major axis, perigee radius (eccen-

tricity), and inclination. This provides control of the secular RAAN rate for the chasers. As with the anchor, there is no direct control of the RAAN, the argument of perigee, or the longitude.

To explore the tradeoffs in satellite constellation design, we employ the Borg multiobjective evolutionary algorithm (MOEA) [14]. The choice of a heuristic optimization approach is driven by the difficulty of the search problem, with the possibility for highly nonlinear and discontinuous mapping between the decision variables and the objective function. This study builds on prior studies using the Borg algorithm and its predecessors for the satellite constellation design problem [15,16]. The Borg MOEA improves upon previous algorithms by combining multiple operators probabilistically to navigate the search space. Operators are selected adaptively based on their demonstrated probability of improvement during the optimization. Borg also integrates several recent advances in the evolutionary algorithms literature, including epsilon-dominance archiving [5], adaptive population sizing [17], and a steady-state algorithm structure [18]. Relative to other MOEAs, Borg has shown superior performance on challenging nonlinear, nonconvex, multimodal problems [19–21].

The Borg algorithm is used to find the Pareto-approximate (or nondominated) set of solutions for each problem formulation. These solutions are not inferior in any objective with regard to all other feasible solutions[22]. The term Pareto-approximate reflects the fact that the optimal set of solutions remains unknown, but we aim to provide as close an approximation as possible. The results reported here were obtained from search evaluations of over 5 million simulated orbital designs. High-fidelity orbit propagation and careful simulation of station-keeping significantly

increased the computational demands of simulating each candidate design. More details on the simulation-optimization framework can be found in [15].

Supplementary Table 4 summarizes the search domain of orbital elements for the 48-hour period search case. The semi-major axis, eccentricity, and inclination search spaces all employ an anchor satellite paradigm whereby the selection of these elements for the first satellite (Sat 1) drives the selection of the elements for the remaining satellites. The semi-major axis search domain varies slightly from the ideal 48-hour value so that the MOEA could explore the impact of variation of the initial conditions (i.e., harness the energy in orbital perturbation forces). The inclination of the anchor spacecraft varies from 5-175 degrees in the 48-hour case, and the inclination of the remaining satellites vary by $\pm 2.5°$ from the anchor value. Limiting the satellite-to-satellite difference in inclination restricts the search space to those constellations which would have similar launch requirements for each vehicle. The RAAN, argument of perigee, and true anomaly at epoch determine the phasing of the orbit planes relative to one another, the position of perigee in each plane, and the initial location of each satellite in the orbit. They all vary over the full range of possible values (0-360), except for the anchor satellite which has a fixed true anomaly at epoch. Fixing one of the true anomalies slightly reduces the size of the search space while still allowing arbitrary relative positioning of the remaining spacecraft.

Eccentricity varies for the anchor vehicle between 0.02988 and 0.8505 in the 48-hour period case. The eccentricity of the remaining satellites individually vary from the anchor value by up to 0.01494. This value constrains the eccentricity search domain such that the resulting minimum

Supplementary Table 3: Allowable ranges of orbital elements of the lifetime simulation around the nominal value.

Element	Station-keeping Range
Semi-Major Axis	± 100 km
Perigee Radius	± 1000 km
Inclination	$\pm 2.5°$

Supplementary Table 4: Orbital element search domain for the 48-hour study.

Element	Sat 1	Sat 2	Sat 3	Sat 4
a (km)	66,927-66,935	a_1	a_1	a_1
e	0.02988-0.8505	$e_1 \pm 0.01494$	e_2	e_2
i (deg)	5-175	$i_1 \pm 2.5$	$i_1 \pm 2.5$	$i_1 \pm 2.5$
Ω (deg)	0-360	0-360	0-360	0-360
ω (deg)	0-360	0-360	0-360	0-360
ν_0 (deg)	180	0-360	0-360	0-360

value of eccentricity constrains the difference between the radii of apogee and perigee to at least 2,000 km. The maximum value of eccentricity likewise limits the radius of perigee value to at least 9,000 km. These ranges avoid restricted regions of the search space where the decision space configuration provided to the MOEA could lead to selections of eccentricity less than zero, while still providing a large search space for the MOEA. Supplementary Figure 4 illustrates the breadth of the range of eccentricity available to the MOEA for exploration, and provides orbit radius values at significant points in each orbit. Despite the lower-bound restriction on eccentricity, the search range provides the MOEA with the option to select effectively circular orbits. Configuring the problem formulation in this way alongside the station-keeping strategy provides the MOEA with a large decision space that yields sufficient flexibility to allow it to bias the initial state of the constellation to allow the perturbing forces to drift the constellation through a nominal state over its lifetime.

The 24-hour period case is summarized in Supplementary Table 5 and incorporates lessons learned from trends identified during the 48-hour search. The semi-major axis is given more freedom to vary from the ideal 24-hour value to better quantify the desirability of groundtrack repeatability. As with the 48-hour case, the MOEA converged to the ideal 24-hour value. The allowable initial eccentricity for the anchor satellite ranges from 0.02 to 0.7628, and the eccentricities of the remaining satellites were allowed to vary 0.01 from the anchor value. As with the 48-hour case, these ranges preclude the eccentricities from entering restricted ranges. The 48-hour case did not generate retrograde orbiting solutions of interest, so the component of the inclination search space greater than 90 degrees is not included in the 24-hour case. RAAN, argument of perigee, and true

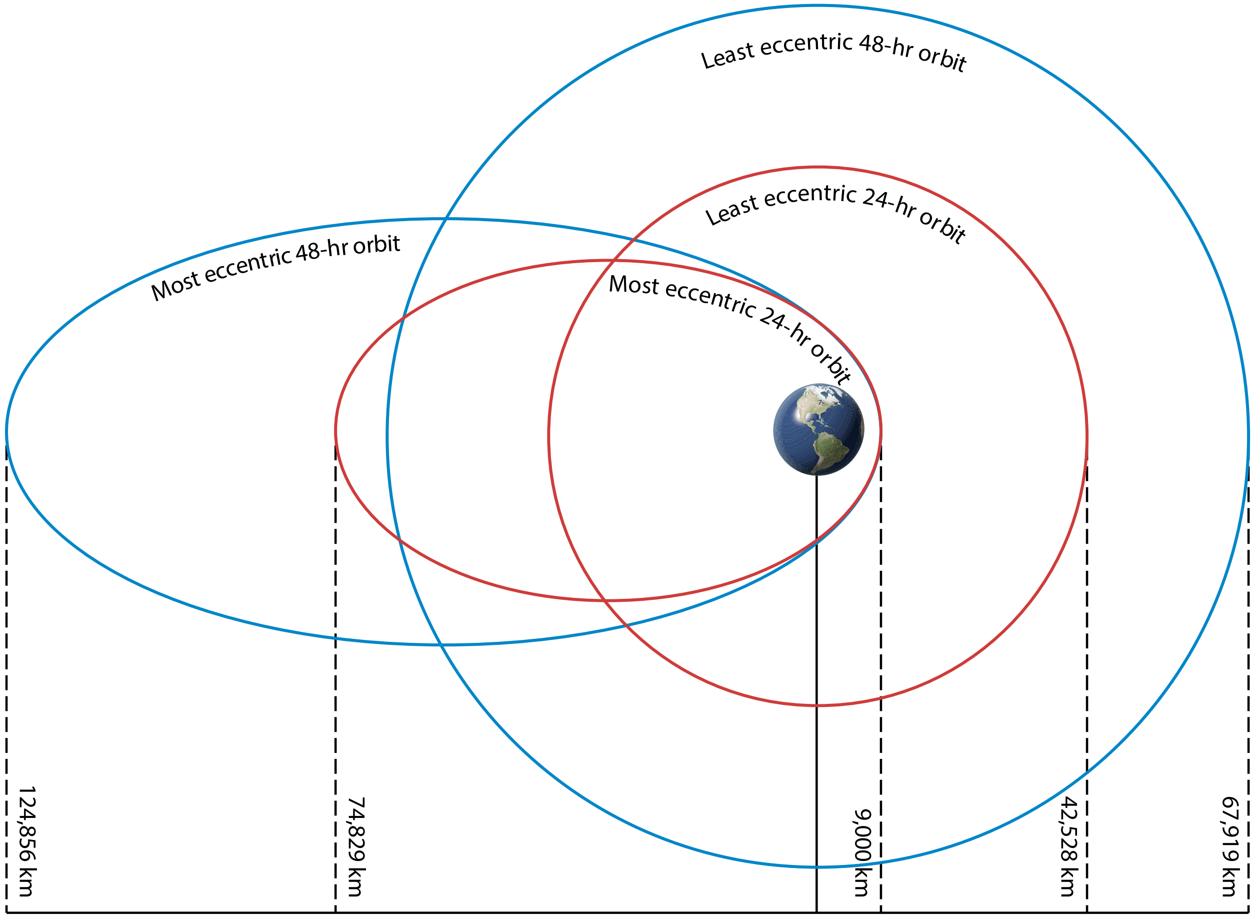

Supplementary Figure 4: Least and most eccentric orbits possible for the 24- and 48-hour studies. Dashed lines indicate orbit radii at significant points. In addition to a broad range of eccentricities, the MOEA could explore a wide range of inclinations, RAANs, arguments of perigee, and true anomalies at epoch.

anomaly variables all vary over the full available range. For both 24- and 48-hour cases, the field of view of each spacecraft is the area under the spacecraft with elevation angle greater than $0°$; this carries with it an implicit assumption neglecting any terrain blockage.

Supplementary Table 5: Orbital element search domain for the 48-hour study.

Element	Sat 1	Sat 2	Sat 3	Sat 4
a (km)	42,114-42,214	a_1	a_1	a_1
e	0.02-0.7628	$e_1 \pm 0.01$	$e_1 \pm 0.01$	$e_1 \pm 0.01$
i (deg)	5-90	$i_1 \pm 2.5$	$i_1 \pm 2.5$	$i_1 \pm 2.5$
Ω (deg)	0-360	0-360	0-360	0-360
ω (deg)	0-360	0-360	0-360	0-360
ν_0 (deg)	180	0-360	0-360	0-360

3 Supplementary References

1. Draim, J. E. Three-and four-satellite continuous-coverage constellations. *J. Guid. Control Dynam.* **8**, 725–730 (1985).

2. Draim, J. A common-period four-satellite continuous global coverage constellation. *J. Guid. Control Dynam.* **10**, 492–499 (1986).

3. Chao, C. Long-term orbit perturbations of the draim four-satellite constellations. In *Proc. AAS/AIAA Astrodynamics Specialist*, 208–213 (1990).

4. Chao, C. Long-term orbit propagations of the draim four-satellite constellations. *J. Guid. Control Dynam.* **15**, 1406–1410 (1992).

5. Laumanns, M., Thiele, L., Deb, K. & Zitzler, E. Combining convergence and diversity in evolutionary multiobjective optimization. *Evol. Comput.* **10**, 263–282 (2002).

6. Brouwer, D. & Clemence, G. M. *Methods of Celestial Mechanics* (Elsevier, 2013).

7. Chao, C.-C. . & Hoots, F. *Applied Orbit Perturbation and Maintenance, Second Edition* (American Institute of Aeronautics and Astronautics, Inc., 2017). URL http://dx.doi.org/10.2514/4.989278.

8. Prince, P. J. & Dormand, J. R. High order embedded Runge-Kutta formulae. *J. Comput. Appl. Math* **7**, 67–75 (1981).

9. Dormand, J. & Prince, P. Runge-Kutta-Nystrom triples. *Comput. Math Appl.* **13**, 937–949 (1987).

10. Montenbruck, O. & Gill, E. *Satellite Orbits: Models, Methods, Applications* (Springer, New York, 2nd ed., 2001).

11. Hairer, E., Nørsett, S. P. & Wanner, G. Solving Ordinary Differential Equations I: Nonstiff Problems (1993).

12. Pines, S. Uniform representation of the gravitational potential and its derivatives. *AIAA J.* **11**, 1508–1511 (1973).

13. Vallado, D. A. *Fundamentals of Astrodynamics and Applications*, vol. 12 (Springer Science & Business Media, 2001).

14. Hadka, D. & Reed, P. Borg: An auto-adaptive many-objective evolutionary computing framework. *Evol. Comput.* **21**, 231–259 (2013).

15. Whittecar, W., DiPrinzio, M., Singh, L., Ferringer, M. & Reed, P. Petascale discovery of passively controlled satellite constellations for global coverage. In *Proc. AAS/AIAA Astrodynamics Specialist* (2015).

16. Reed, P. *et al.* Internationally coordinated multi-mission planning is now critical to sustain the space-based rainfall observations needed for managing floods globally. *Environ. Res. Lett.* **10**, 024010 (2015).

17. Kollat, J. B. & Reed, P. M. A computational scaling analysis of multiobjective evolutionary algorithms in long-term groundwater monitoring applications. *Adv. Water Resour.* **30**, 408–419 (2007).

18. Deb, K., Mohan, M. & Mishra, S. Evaluating the ε-domination based multi-objective evolutionary algorithm for a quick computation of pareto-optimal solutions. *Evol. Comput.* **13**, 501–525 (2005).

19. Hadka, D. & Reed, P. Diagnostic assessment of search controls and failure modes in many-objective evolutionary optimization. *Evol. Comput.* **20**, 423–452 (2012).

20. Hadka, D., Reed, P. M. & Simpson, T. W. Diagnostic assessment of the borg moea for many-objective product family design problems. In *IEEE C. Evol. Computat.*, 1–10 (IEEE, 2012).

21. Reed, P. M., Hadka, D., Herman, J. D., Kasprzyk, J. R. & Kollat, J. B. Evolutionary multiobjective optimization in water resources: The past, present, and future. *Adv. Water Resour.* **51**, 438–456 (2013).

22. Woodruff, M. J., Reed, P. M. & Simpson, T. W. Many objective visual analytics: rethinking the design of complex engineered systems. *Struct. Multidiscip. O.* **48**, 201–219 (2013).

Correspondence Correspondence and requests for materials should be addressed to L.A.S. (email: lake.a.singh@aero.org).
